# Trail-blazing and keeping pace: building, retaining and expanding image analysis expertise

**DOI:** 10.3389/fbinf.2025.1613866

**Published:** 2025-05-30

**Authors:** David Kirchenbuechler, Mariana De Niz, Constadina Arvanitis

**Affiliations:** ^1^ Center for Advanced Microscopy and Nikon Imaging Center, Feinberg School of Medicine, Northwestern University, Chicago, IL, United States; ^2^ Department of Cell and Developmental Biology, Feinberg School of Medicine, Northwestern University, Chicago, IL, United States

**Keywords:** microscopy, bioinformatics, facilities, research, analysis

## Abstract

Scientific studies are increasingly complex, involving quantification of many different experimental approaches and technologies. However, it is challenging for any individual scientist to build and retain sufficient expertise and competency in a large range of scientific tools. A deep expertise is critical for rigor and reproducibility; however, focused expertise can easily become a hindrance to inter-disciplinary science. This is particularly true with respect to microscopy and image analysis. Core facilities often bridge this gap, serving as an access point to expertise in cutting-edge technologies while facilitating collaboration. Our purpose with this perspective piece is to share our experience with other Microscopy Core Facility Directors and Image analysts who are aiming to establish image analysis training as a service. We hope that this shared experience can help others optimize their service though our lessons learned, and avoid pitfalls we faced during our Core’s timeline. In this paper we explore three elements that have been vital for the establishment and expansion of image analysis at the Center for Advanced Microscopy at Northwestern University. The first is a commitment to dedicated image analysis service. The second is establishing image analysis training programs for the local scientific community, which facilitates integration of analysis into microscopy workflows. The third is engagement with international organizations such as BINA. These organization foster collaborations which ultimately result in the fruitful dissemination of novel tools across the community. These three elements are essential to maximize the potential of imaging-based scientific research and ultimately ensuring equal access to image informatics.

## Introduction

Core facilities play a vital role in centralizing and democratizing cutting-edge technology and allowing access to highly skilled personnel who can guide researchers in the use of most current methods and instrumentation. Imaging technologies have become more sophisticated and specialized image analysis expertise has become a critical component of microscopy workflows. In this work, we focus on the establishment and evolution of image analysis services at the Center for Advanced Microscopy (CAM) at Northwestern University. This is with the purpose of sharing this experience with other Core Directors who might benefit from our lessons learned. CAM’s image analysis services have consolidated into three key elements. First, we made a commitment to go beyond consultations and generate customized workflows and subsequent adaptive support for users; this has become the definition of our image analysis services. Second, we developed programs to educate and train our local community and users. Third, we have integrated into the international community through organizations such as BINA to promote bilateral cooperation and exchange of expertise. Ultimately, we discuss the impact that these steps have had on our local Northwestern University community, from ensuring scientific reproducibility to facilitating the acquisition of in-depth expertise and scientific independence.

## Discussion

### Commitment to dedicated image analysis service

**FIGURE 1:**
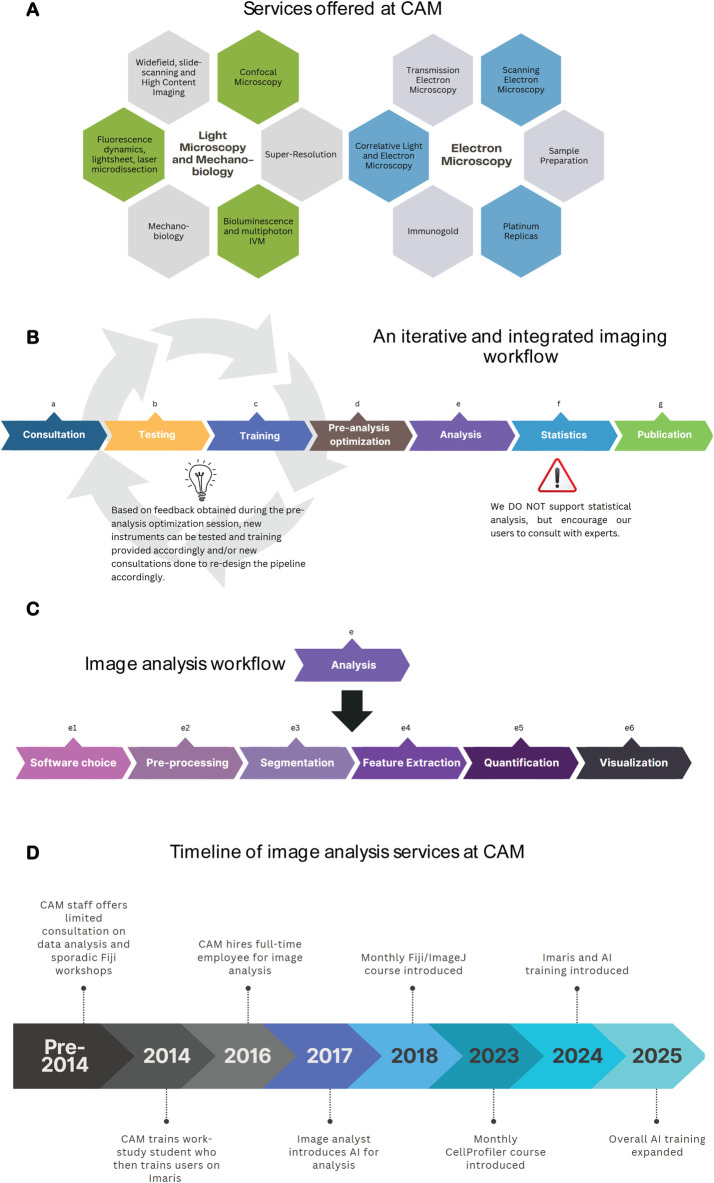
CAM’s commitment to dedicated image analysis services. **(A)** CAM offers 40 service lines, encompassing light microscopy, electron microscopy, mechanobiology and image analysis. **(B)** CAM training workflow a) Consultation: discuss project goals and define the techniques and instruments to use; b) Testing (optional): used for non-standard imaging to test various instruments and/or techniques; c) Training: encompasses two training sessions; d) Pre-analysis optimization: consult with image analyst to determine how if images acquired are suitable for downstream analysis; e) Analysis: See Figure 1C; f) Statistics: seek statistical support from appropriate experts; g) Publication: Support manuscript preparation, focus is often on materials and methods and acknowledgements sections **(C)**. Analysis workflow at CAM: Routine user analysis protocols encompass advice on the suitable software choice, pre-processing methods (including filtering, denoising, etc.), segmentation (different strategies for doing so depending on the research question), feature extraction, quantification and visualization. **(D)** Timeline of key milestones achieved by CAM since its creation to date in terms of image analysis. Figures were created with Canva and/or Biorender.

CAM was founded in 2002, and it became a Nikon Imaging Center (NIC) in 2008. Nikon Imaging Centers are state-of-the-art imaging facilities established as partnerships between various institutes around the world, and Nikon. Since its creation, CAM has continuously expanded its services (Figure 1A) by increasing equipment and staffing levels. Each of these tools requires specialized expertise, which we have divided between our team. These approaches yield a variety of images that require unique workflows for analysis. As CAM’s offerings grew in complexity and the user base expanded, it became clear that the staff lacked the bandwidth to support image analysis needs. This was first remedied by the hiring of a work study student to help support Imaris workstation training, but by 2016 the demand and experimental complexity was too high for this level of support and our core established a dedicated high level ‘Image Analyst’ position.

Since its inception, the concept of this position has evolved to adapt to our user needs. Key lessons we have learned along the way include.

1. An iterative and integrated imaging workflow: Constant communication between the user, CAM microscopy staff and the image analysts is critical to ensure the highest quality data and workflow (Figure 1B). The most efficient workflow begins with an initial user consultation. This is an in-depth discussion about their research questions and imaging needs where we advise on sample preparation considerations and identify the suitable instrument. This is followed by training at the microscope where staff emphasize essential concepts that impact data quality. Examples include numerical aperture, pinhole size, pixel/voxel size, saturation, etc., with a CAM-provided training sample. A second training session allows the researcher to define acquisition parameters with their samples. This is performed with a limited number of samples and images to decide, together with the image analyst, whether the acquisition parameters are suitable for downstream analysis, and relevant to the research question. This is often an iterative process, until data acquisition and analysis are streamlined to the point where a user can proceed independently. Having established a relationship with CAM staff they are more comfortable seeking further guidance at any point of their work. When followed, this method has helped reinforce the importance of key concepts, streamline image analysis, reduce monetary costs for users, and avoid unnecessary time investment in acquiring images at the wrong instruments, or with inadequate acquisition parameters.
2. How we support image analysis service and their boundaries. It became apparent almost immediately after establishing a data analysis position, that performing the analysis on behalf of users is not sustainable or scalable. Additionally, because Northwestern University is an academic institution, we have made education a key part of CAM’s mission and believe that it is essential for scientists to fundamentally understand how their data has been analyzed when they draw their scientific conclusions. In order to accomplish this in the most efficient manner we include a subsequent consultation and separate workflow specifically for image analysis training. This workflow pinpoints the research needs, identifies the approach and software required, and defines an initial analysis protocol (Figure 1C). The protocols can be on a variety of different software packages depending on the analysis needs. Our facility uses a combination of commercial (e.g., NIS Elements, TissueQuest Imaris) and open-source software packages (e.g., Fiji, CellProfiler, Napari, QuPath, Ilastik, PhasorPy) and provides continuous guidance on acquired images. We do not provide statistical services for tasks prior to image acquisition (i.e., to calculate statistical power of how many images are required to demonstrate a specific phenotype), nor to define statistical significance of the resulting image analysis. Northwestern University, however, has two core facilities that offer such services, and which we encourage our users to seek help from.
3. ‘Simpler is better’. While learning how to best support user needs, initial workflows were more complex, including the use of programming in MATLAB. However, our team quickly realized that simpler workflows with user-friendly GUIs fostered user confidence and autonomy to carry out the analysis. These software packages allowed users to build-up their image analysis expertise without the barrier of understanding complex computer programs. At CAM we consider it vital that our users, as scientists, understand what is behind the images they acquire, and we found that the best way to achieve this is to encourage a hands-on approach as opposed to us providing publication-ready results. This is as true for image analysis as it is for imaging.

We give an overview of the evolution of image analysis at CAM in Figure 1D. Currently, approximately 50% of the laboratories who use our facility for light microscopy utilize image analysis services. In the past year alone, we have supported over 200 individual users with image analysis, from a wide range of Departments and disciplines. Our approach to teaching, as opposed to performing the project’s image analysis ourselves, has resulted in increased proficiency by our users and streamlined support for them.

Considerations for the future of image analysis at CAM include the following three directions.

1. Our analyst position has been established, and demand is constantly increasing. There are two ways that we hope to expand image analysis services in the future. One is by educating our existing staff members to better support image analysis at the user level. The second is to create avenues such as internship programs to train future image analysis specialists and potentially bring in new staff members.
2. Image analysis is a relatively new field that is constantly evolving. This is most evident with the advent of deep learning and other AI-based image analysis tools. The ability to navigate proper use of AI (generating adequate controls, training and documentation) is currently not well established and doing so is of the utmost importance to ensure the validity of scientific conclusions, and the reproducibility of results (Hill et al., 2024). Knowledge on best practices must be integrated into user training and education.
3. Many imaging centers lack powerful computational resources. Advancements in instrument development is generating bigger datasets and core facilities need adequate computing power, workflows and data management in place. Analysis of these datasets is moving from individual computers to consolidated computing resources such as cluster and cloud computing. We expect to expand our capacities to do the same. This also has the added advantage of enabling researchers to access data and analyze it from anywhere.

Ultimately, at CAM we are convinced of the relevance of the image analyst position and the positive contributions it has had to the scientific community. This enables us to continue to build a state-of-the-art toolkit for image analysis as this discipline continues to evolve to allow novel possibilities for scientific research.

#### Establishment of image analysis education at Northwestern University

In addition to the one-on-one support for specific projects, we identified the need to build our researchers’ image analysis knowledge base via educational initiatives. We have done so in three different ways.

**FIGURE 2:**
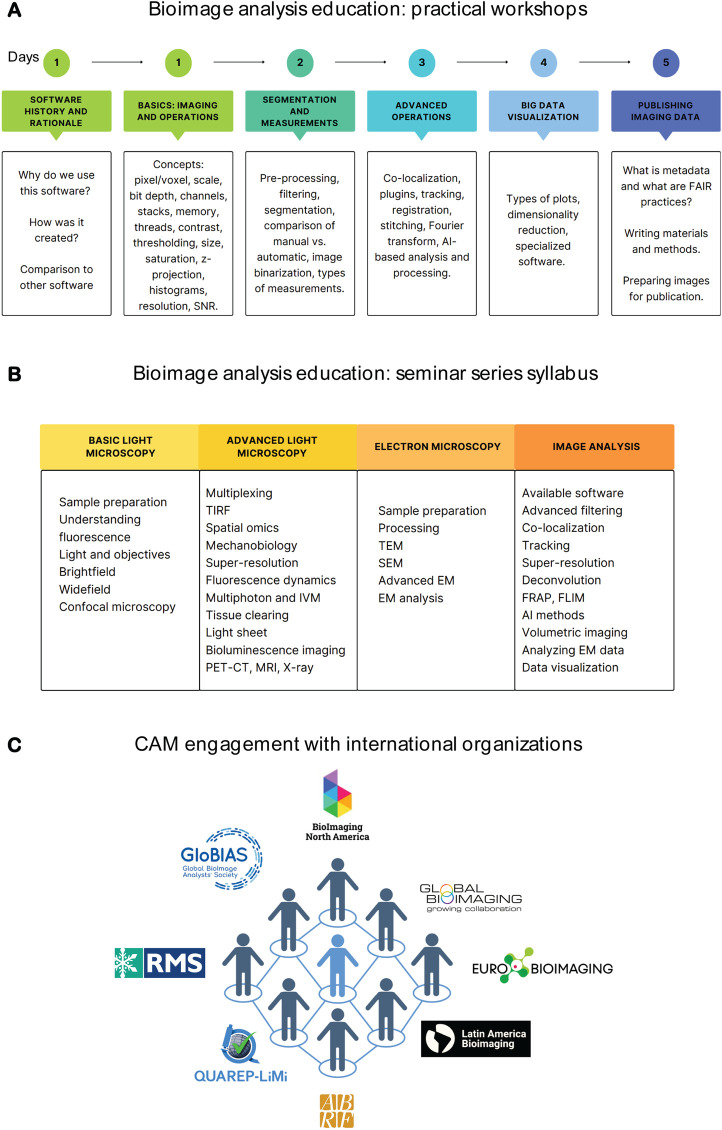
CAM’s efforts towards education and international collaboration. **(A)** Syllabus overview of CellProfiler and Fiji workshops. **(B)** Syllabus of the yearly CAM seminar series. **(C)** CAM’s engagement with international organizations. Figures were created with Canva and/or B.iorender.

1. General education. We give 1–2 h lectures in different graduate programs, where we cover key concepts on images, processing and analysis. Although this is a brief introduction, it is an important part of student education because it emphasizes that analysis is an integral part of imaging workflows. It also is a starting point for students to connect to core facility staff for future help with imaging analysis workflows.
2. Practical workshops. We have found that practical workshops are vital to expanding user knowledge and understanding of workflows and key concepts. These workshops allow researchers hands-on experience with software, and ultimately teaching them how to build workflows using validated real world examples. We have been performing these workshops since 2013 and currently offer 10-h practical Fiji or CellProfiler workshops, alternating monthly. Ideally, these courses should be offered at regular intervals which allow new imaging scientists the opportunity to access them and not just once a year. We chose these two software packages because they are open source, run on various operating systems, are easily accessible to users, and most importantly have robust online support communities with new features being added constantly. We routinely incorporate new developments such as AI processing into our workshops. Both workshops follow similar workflows, and their content is summarized in Figure 2A. In addition, we organize yearly courses for our commercial platforms in which we host industry representatives to give an in-depth workshop on basic and novel features of their software. Feedback from these workshops allows us to identify user needs and as a result we are able to incorporate these suggestions into a seminar series dedicated to specialized topics of imaging and image analysis.
3. Advanced image analysis topics. Through user consultations and workshop feedback we have been able to identify specific needs for in-depth topics and organized a seminar series focusing on a single topic at a time. Examples of these seminars are highlighted in Figure 2B. In general, these seminars are more lecture-focused and do not include hands-on practical exercises so that the lecture can be software agnostic. As a bonus, the materials generated for these topics are then used when we have consultations with users who are interested in performing specific analyses.

So far, our initiatives for image analysis education have been well-received by the research community at Northwestern University. The vast majority of our users seek to deepen their understanding of image analysis and go on to become confident in this knowledge in a routine manner.

#### Engagement with national and international organizations

When we first established a full-time image analysis position we were largely isolated from the international community and relied mostly on surveilling publications and online materials to determine optimal workflows. This is challenging for an individual to navigate independently. Over the last decade, several developments have been vital to accelerate the exchange of information and the advancement of image analysis. Critical among them are international organizations that bring together image analysts from around the globe to share expertise and generate resources in an efficient and collaborative manner. These organizations have had a significant impact on the further evolution of image analysis at CAM.

We currently are part of multiple local, national and international communities. Internationally, CAM members are part of BioImaging North America (BINA), Global Bioimaging (GBI), Latin America Bioimaging (LABI), Global BioImaging Analysts Society (GloBIAS), and QUAREPLiMi (Quality Assessment and Reproducibility for Instruments & Images in Light Microscopy). Nationally we are part of the Association of Biological Research Facilities (ABRF) and the American Society for Cell Biology (ASCB). Locally, we have formed a Microscopy Journal Club that involves institutions in the Chicagoland area, where we foster inter-institutional collaborations. Our participation in these organizations and initiatives has resulted in a bidirectional flow of expertise (Figure 2C). Currently, participation is fully by CAM staff; nevertheless, faculty members and trainees are always included in initiatives we lead, with several being speakers at BINA Exchange of Experience and Speaker Series, and with many participating in the Chicagoland journal club. Moreover, we advertise relevant activities and promote connecting with national and international organizations through CAM’s seasonal newsletter, distributed to all Northwestern researchers.

Through the international organizations of which we are members and contributors, we have had the opportunity to expand our expertise in various ways. The Professional Development Awards given by BINA have funded our team’s participation in several training programs. These include the Advanced Fluorescence Imaging and Dynamics workshop at the Laboratory of Fluorescence Dynamics (LFD) at UC Irvine, the Lightsheet Fluorescence Microscopy (LSFM) course at Woods Hole, the Tissue Clearing course at MicRoN, Harvard University, and the Machine Learning and the Bioimage Archive workshop at EMBL-EBI. These courses have impacted our users directly. For example, we now fully support FLIM analysis, and big data processing, including deep learning, for data obtained from lightsheet microscopy. Moreover, we have held important conversations about the future of data management.

Our team’s participation at BINA, LABI, GBI, ABRF and ASCB meetings has resulted in a positive exchange of ideas with other experts in the field. Examples of significant contributions resulting from these exchanges have been the implementation of the practical training workshop on CellProfiler and CellProfiler Analyst (Carpenter et al., 2006; McQuin et al., 2018; Stirling et al., 2021; Weisbart et al., 2024), the incorporation of training on computational methods for super-resolution such as MSSR (Torres-García et al., 2022), and the furthering of artificial intelligence at CAM. BINA has also facilitated events where our team has had the chance to interact with QUAREPLiMi members to discuss and implement the most current quality control practices and metrics (Boehm et al., 2021; Nelson et al., 2021), and to learn about the state-of-the-art teaching methods and norms regarding metadata handling.

Through BINA and GBI, the CAM team has also had the chance to engage in translation of teaching material to other languages, including Dr. Beth Cimini’s ‘Bioimaging Guide’ (Senft et al., 2023; Cimini, 2024), Dr. Peter Bankhead’s, ‘Introduction to Bioimage Analysis’ book (Bankhead, 2024). These activities are consistent with our aim of democratizing access to microscopy resources, and we refer researchers are Northwestern University to all the original and translated versions of these resources, to complement their knowledge on image analysis. This engagement has also led to collaborations with other organizations, such as RMS and LABI, for breaking language barriers to access microscopy and image analysis expertise.

BINA and ABRF have provided platforms for us to reach out to an international audience to discuss artificial intelligence-leveraged microscopy and image analysis. At a BINA Exchange of Experience seminar organized by the Image Informatics group, we shared our experience on implementing artificial intelligence for imaging and image analysis at the core facility at three key steps of the imaging workflow: image acquisition, image analysis, and data evaluation. Equally, ABRF gave us the opportunity to engage in multiple open seminars and practical workshops focusing on AI, its relevance to image analysis, and important considerations for its use and implementation in core facilities. These collaborations have formed the foundation of support from an external community for image analysis within a core.

## Conclusion

Our commitment to supporting researchers on image analysis has evolved from opening a specific position dedicated to image analysis, to developing a plethora of tools for the benefit of our research community over the last decade. This includes one-on-one consultations, open hours, lectures, practical workshops, and seminar series. More recently, we have increased our efforts to connect with the international image informatics community, which has allowed us to establish bilateral cooperation, and to increase our local expertise with state-of-the-art tools in this rapidly evolving discipline. BINA has been instrumental in this process, fostering, funding and facilitating international collaborations, and connecting experts worldwide. We look forward to continuing our work together, and consider this model of cooperation an example for the future of microscopy and image informatics. We hope this perspective and lessons learned (summarized in Supplementary Figure S1), will serve as useful guidance for Core Directors and image analysts aiming to establish and/or grow image informatics services in their facility.

## Data Availability

The original contributions presented in the study are included in the article/Supplementary Material, further inquiries can be directed to the corresponding author.
