# Supplementary material for: Trail-blazing and keeping pace: building, retaining and expanding image analysis expertise
Source: Front Bioinform. 2025 May 30;5:1613866. doi: 10.3389/fbinf.2025.1613866 (PMC12162573; doi:10.3389/fbinf.2025.1613866)
Supplement: Supplementary file 1 [file DataSheet1.pdf]

Generalized Image Analysis Implementation Roadmap

1. PROOF OF CONCEPT

Convince your university of the value of image analysis. Interns, work-study trainees or part-time core members can cover this work.

2. DEDICATED POSITION

Garnering enough interest from an institutional point of view is key to fund a dedicated person or group for image analysis.

3. DEFINE THE SERVICE YOU WILL PROVIDE

Define the service line to avoid ambiguity:

- Do you perform image analysis for researchers?
- Do you train researchers to do their own analysis?

4. IDENTIFY USER NEEDS

Identify the main image analysis needs of your research community and begin creating and familiarizing yourself with the type of analysis that would most benefit them.

5. SIMPLER IS BETTER

In general, do not overcomplicate imaging workflows or training programs. Simpler workflows enable better learning outcomes.

6. CREATE TRAINING PROGRAMS

Tailor your educational programs to your user needs. We suggest three different lines:

- Lecture series covering theory
- Hands-on workshops in small groups to practice
- One-on-one consultations to tailor the workflow

7. JOIN THE INTERNATIONAL COMMUNITY

Do this as soon as possible after creating a dedicated position. Avoid being isolated. Many solutions already exist. Connecting with the international community enables sharing knowledge and information.

8. EXPAND EXPERTISE

Identify novel technologies and image analysis methods and tools to keep your services state-of-the-art and at the vanguard of research

9. ENSURE SUSTAINABILITY

If your user needs grow in complexity and demand, make sure you are able to provide them accordingly. Prepare to avoid bottlenecks.
